# Prostate cancer in multi‐ethnic Asian men: Real‐world experience in the Malaysia Prostate Cancer (M‐CaP) Study

**DOI:** 10.1002/cam4.4319

**Published:** 2021-10-09

**Authors:** Jasmine Lim, Rohan Malek, Sathiyananthan JR, Charng C. Toh, Murali Sundram, Susan Y. Y. Woo, Noor A. M. Yusoff, Guan C. Teh, Benjamin J. T. Chui, Ing S. Ngu, S. Thevarajah, Wei J. Koh, Say B. Lee, Say C. Khoo, Boon W. Teoh, Rohana Zainal, Teck M. Tham, Shamsuddin Omar, Noor A. Nasuha, Hideyuki Akaza, Teng A. Ong, M‐CaP Study

**Affiliations:** ^1^ Department of Surgery Faculty of Medicine University of Malaya Kuala Lumpur Malaysia; ^2^ Department of Urology Selayang Hospital Ministry of Health Malaysia Selangor Malaysia; ^3^ Department of Urology Kuala Lumpur Hospital Ministry of Health Malaysia Kuala Lumpur Malaysia; ^4^ Department of Urology Sarawak General Hospital Ministry of Health Malaysia Sarawak Malaysia; ^5^ Department of Urology Queen Elizabeth Hospital Ministry of Health Malaysia Kota Kinabalu Malaysia; ^6^ Department of Urology Penang Hospital Ministry of Health Malaysia Penang Malaysia; ^7^ Department of Surgery Sultanah Bahiyah Hospital Ministry of Health Malaysia Alor Setar Malaysia; ^8^ Department of Urology Sultanah Aminah Hospital Ministry of Health Malaysia Johor Bahru Malaysia; ^9^ Department of Surgery Raja Perempuan Zainab II Hospital Ministry of Health Malaysia Kota Bahru Malaysia; ^10^ Strategic Investigation on Comprehensive Cancer Network Interfaculty Initiative in Information Studies / Graduate School of Interdisciplinary Information University of Tokyo Tokyo Japan

**Keywords:** Asia, advanced prostate cancer, cancer care disparities, cancer registry, The A‐CaP Study Group

## Abstract

Prostate cancer is the third most common cancer in Malaysia with the lifetime risk of 1 in 117 men. Here, we initiated a longitudinal Malaysia Prostate Cancer (M‐CaP) Study to investigate the clinical and tumour characteristics, treatment patterns as well as disease outcomes of multi‐ethnic Asian men at real‐world setting. The M‐CaP database consisted of 1839 new patients with prostate cancer diagnosed between 2016 and 2018 from nine public urology referral centres across Malaysia. Basic demographic and clinical parameters, tumour characteristics, primary treatment, follow‐up and vital status data were retrieved prospectively from the hospital‐based patients’ case notes or electronic medical records. Primary endpoints were overall survival (OS) and biochemical progression‐free survival (bPFS). The median age at diagnosis of M‐CaP patients was 70 years (interquartile range, IQR 65–75). Majority of patients were Chinese (831, 45.2%), followed by Malays (704, 38.3%), Indians (124, 6.7%) and other races (181, 9.8%). The median follow‐up for all patients was 23.5 months (IQR 15.9–33.6). Although 58.1% presented with late‐stage cancer, we observed ethnic and geographic disparities in late‐stage prostate cancer diagnosis. Curative radiotherapy and primary androgen deprivation therapy were the most common treatment for stage III and stage IV diseases, respectively. The median OS and bPFS of stage IV patients were 40.1 months and 19.2 months (95% CI 17.6–20.8), respectively. Late stage at presentation remains a challenge in multi‐ethnic Asian men. Early detection is imperative to improve treatment outcome and survival of patients with prostate cancer.

## Graphical abstract

Late stage at presentation remains a challenge in multi‐ethnic Asian men. We observed ethnic and geographic disparities in late stage prostate cancer diagnosis. Early detection is imperative to improve treatment outcome and survival of prostate cancer patients.

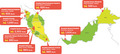

## 1 INTRODUCTION

Prostate cancer is the fifth most commonly diagnosed cancer and the seventh leading cause of cancer mortality in Asia.^1^ Important cancer mortality‐to‐incidence ratio differences exist in prostate cancer epidemiology among Asian countries. Comparing with Japan and South Korea, developing Southeast Asian countries including Philippines, Malaysia and Thailand recorded more than twofold higher mortality‐to‐incidence ratio.^2^ These discrepancies indicate inequalities in cancer survival rates across high and low‐to‐middle‐income countries.^3^

With the increase of aging population, urbanisation and westernised lifestyle, one can expect a rapid growing trend of prostate cancer incidence in Asian men in the near future. It is imperative to improve our understanding of prostate cancer in Asia for better cancer control planning and reduced cancer burden in the community. The Japanese Study Group of Prostate Cancer (J‐CaP) was established as a nationwide longitudinal prospective cohort study in 2001 to assess the outcomes of prostate cancer patients undergoing androgen deprivation therapy (ADT).^4^ In 2015, the United in Fight against Prostate Cancer (UFO) registry was commenced focusing on the management and patient‐reported quality of life of advanced prostate cancer in eight Asian countries.^5^ The availability of longitudinal, observational prostate cancer database remains scarce in this part of the world.

Therefore, the Asian Prostate Cancer (A‐CaP) Study was initiated as a multicentre, longitudinal prospective cohort study in 2016 involving 12 Asian nations. The registry includes real‐world data from men with newly diagnosed prostate cancer between 2016 and 2018 in Japan, South Korea, China, Singapore, Malaysia, Thailand, Indonesia, Hong Kong, Taiwan, Turkey, Philippines and Vietnam.^6^, ^7^, ^8^, ^9^ The Malaysia Prostate Cancer (M‐CaP) Study Group is one of the key partners in the A‐CaP Study, aiming to evaluate the clinical and tumour characteristics, treatment patterns and survival of prostate cancer patients. Here, we describe an overview of the M‐CaP Study Group and present the baseline disease characteristics, treatment profiles as well as outcome of prostate cancer in multiethnic Southeast Asian men.

## 2 METHODS

### 2.1 Study population

**FIGURE 1:**
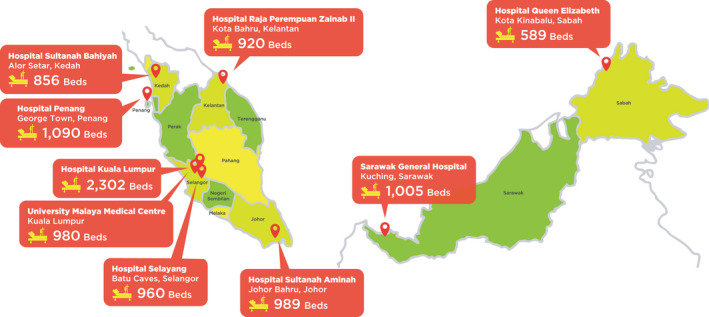
Distribution of all nine public urology referral centers participating in the Malaysia Prostate Cancer (M‐CaP) Study.

The M‐CaP database is a longitudinal study with newly diagnosed patients with prostate cancer between 2016 and 2018 from nine public urology referral centres across eight states in Malaysia. These centres were Hospital Kuala Lumpur, Hospital Selayang, University of Malaya Medical Centre (UMMC), Hospital Penang, Hospital Sultanah Bahiyah, Hospital Sultanah Aminah, Hospital Raja Perempuan Zainab II, Sarawak General Hospital, and Hospital Queen Elizabeth II (Figure 1). The database included basic demographic and clinical parameters, tumour characteristics, primary treatment, follow‐up, and vital status. Data were retrieved prospectively from the hospital‐based patients’ case notes or electronic medical records using a written form (proforma). Details from the proforma were then transferred into the Research Electronic Data Capture (REDCap) System for further analysis. The vital status was obtained annually from the National Registration Department through Biostatics & Data Repository Section, National Institutes of Health (NIH) Malaysia to ensure complete ascertainment of mortality data including date and cause of death.

### 2.2 Variables

Basic demographic data included age at diagnosis (year), ethnicity (Malay, Chinese, Indian and others) and family history of prostate cancer. History of major comorbidities such as hypertension, diabetes, hyperlipidaemia, heart disease and lower urinary tract symptoms, prostate‐specific antigen (PSA) level at diagnosis and Eastern Cooperative Oncology Group status were collected.

Tumour characteristic consisted of histological type, Gleason score grading, primary tumour site (T), lymph node involvement (N) and presence of distant metastasis (M) based on Union for International Cancer Control (UICC) TNM classification. Treatment data included radical prostatectomy, radiotherapy, ADT, chemotherapy and active surveillance or watchful waiting. Role of each treatment was categorised into first‐line curative treatment, neoadjuvant/adjuvant/salvage treatment, primary treatment and palliative treatment. Details of each treatment were collected including type of treatment, start date, end date, lymph node dissection, dosage and reason of termination, as appropriate.

Each newly diagnosed case is followed up closely every 6 months via on‐site data monitoring by trained data abstractors over a minimum duration of 7 years. Subsequent serum PSA levels, TNM restaging, treatment patterns and disease progression were documented prospectively for clinical and biochemical follow‐up. The primary endpoints were overall survival (OS) and biochemical progression‐free survival (bPFS), which were defined as time from the date of diagnosis to death, and time from treatment start date to the first event of PSA recurrence/progression^10^, ^11^ or death, respectively.

### 2.3 Statistical analysis

All categorical variables were described by proportions whilst continuous variables were presented in median and interquartile range (IQR). The median OS and bPFS comparison were estimated with Kaplan–Meier method. All statistical analysis was performed using SPSS for Windows version 21.0 (SPSS Inc.). Two‐tailed *p* value <0.05 was termed as statistically significant.

## 3 RESULTS

A total of 1839 new prostate cancer cases were included into the M‐CaP database. Summary of demographic and clinical characteristics were presented in Table 1. The median age at diagnosis of M‐CaP patients was 70 years (IQR 65–75). Only 8% of patients were aged younger than 60 years at presentation. Majority of patients were of Chinese ethnicity (831, 45.2%), followed by Malays (704, 38.3%), Indians (124, 6.7%), Iban‐Bidayuh (67, 3.6%), Kadazan‐Dusun (38, 2.1%) and other races (75, 4.1%). The M‐CaP patients had a high burden of comorbidity with 52.8% with history of two or more major comorbidities. Family history of prostate cancer was found in 28 (4.1%) cases.

**TABLE 1:** Baseline demographic and clinical characteristics in 1839 patients with prostate cancer.

Characteristics	Frequency distribution, n (%)						
	Overall (N = 1839)	Malay (n = 704)	Chinese (n = 831)	Indian (n = 124)	Iban – Bidayuh (n = 67)	Kadazan‐Dusun (n = 38)	Others (n = 75)
Age at diagnosis, years (median, IQR)	70 (65–75)	69 (64–74)	71 (66–75)	68.5 (64–79.5)	72 (68–75)	70.5 (63.25–75.25)	70 (64–76)
Family history of prostate cancer							
Absent	660 (95.9)	204 (97.1)	314 (94.3)	56 (94.9)	41 (100)	19 (100)	26 (100)
Present	28 (4.1)	6 (2.9)	19 (5.7)	3 (5.1)	0 (0)	0 (0)	0 (0)
*Unknown*	1151	494	498	65	26	19	49
Comorbidity count							
0	457 (24.9)	176 (25.0)	227 (27.3)	21 (16.9)	13 (19.4)	5 (13.2)	15 (20.0)
1	412 (22.4)	149 (21.2)	186 (22.4)	27 (21.8)	20 (29.9)	13 (34.2)	17 (22.7)
2	454 (24.7)	175 (24.9)	201 (24.2)	32 (25.8)	20 (29.9)	11 (28.9)	15 (20.0)
≥3	516 (28.1)	204 (29.0)	217 (26.1)	44 (35.5)	14 (20.9)	9 (23.7)	28 (37.3)
History of LUTS							
Absent	1314 (71.5)	476 (67.6)	620 (74.6)	85 (68.5)	56 (83.6)	24 (63.2)	53 (70.7)
Present	525 (28.5)	228 (32.4)	211 (25.4)	39 (31.5)	11 (16.4)	14 (36.8)	22 (29.3)
ECOG performance status at baseline							
0	456 (55.1)	122 (49.4)	262 (62.2)	33 (64.7)	27 (57.4)	4 (20.0)	8 (19.0)
1	218 (26.3)	69 (27.9)	96 (22.8)	9 (17.6)	16 (34.0)	12 (60.0)	16 (38.1
2	93 (11.2)	31 (12.6)	39 (9.3)	6 (11.8)	1 (2.1)	1 (5.0)	15 (35.7)
3–4	61 (7.4)	25 (10.1)	24 (5.7)	3 (5.9)	3 (6.4)	3 (15.0)	3 (7.1)
*Unknown*	1011	457	410	73	20	18	33
PSA at diagnosis (ng/ml)							
≤10	265 (14.6)	69 (10.0)	157 (19.1)	33 (26.6)	4 (6.0)	0 (0)	2 (2.7)
10.01–20.00	300 (16.5)	92 (13.3)	174 (21.2)	18 (14.5)	7 (10.4)	6 (15.8)	3 (4.0)
20.01–50.00	310 (17.1)	109 (15.8)	148 (18.0)	24 (19.4)	15 (22.4)	5 (13.2)	9 (12.0)
>50	940 (51.8)	420 (60.9)	342 (41.7)	49 (39.5)	41 (61.2)	27 (71.1)	61 (81.3)
*Unknown*	24	14	10	0	0	0	0
Gleason score							
≤6	254 (14.6)	89 (13.2)	126 (15.9)	24 (21.1)	9 (15.5)	2 (5.7)	4 (5.6)
7	534 (30.6)	181 (26.9)	262 (33.1)	37 (32.5)	23 (39.7)	10 (28.6)	21 (29.2)
≥8	956 (54.8)	404 (59.9)	403 (50.9)	53 (46.5)	26 (44.8)	23 (65.7)	47 (65.3)
*Unknown*	95	30	40	10	9	3	3
Presence of metastases (M1)							
No	763 (46.1)	254 (40.6)	410 (54.6)	60 (54.1)	21 (34.4)	4 (10.8)	14 (20.3)
Yes	892 (53.9)	372 (59.4)	341 (45.4)	51 (45.9)	40 (65.6)	33 (89.2)	55 (79.7)
*Unknown*	184	78	80	13	6	1	6
UICC staging system							
Stage I	169 (9.5)	62 (9.0)	89 (11.0)	12 (9.9)	4 (6.5)	0 (0)	2 (2.8)
Stage II	326 (18.2)	109 (15.8)	169 (20.9)	27 (22.3)	9 (14.5)	2 (5.3)	10 (14.1)
Stage III	254 (14.2)	87 (12.6)	144 (17.8)	13 (10.7)	5 (8.1)	3 (7.9)	2 (2.8)
Stage IV	1039 (58.1)	430 (62.5)	406 (50.2)	69 (57.0)	44 (71.0)	33 (86.8)	57 (80.3)
*Unknown*	51	16	23	3	5	0	4
Year of diagnosis							
2016	560 (30.4)	224 (31.8)	240 (28.9)	47 (37.9)	22 (32.8)	9 (23.7)	18 (24.0)
2017	601 (32.7)	214 (30.4)	295 (35.5)	37 (29.8)	23 (34.3)	9 (23.7)	23 (30.7)
2018	678 (36.9)	266 (37.8)	296 (35.6)	40 (32.3)	22 (32.8)	20 (52.6)	34 (45.3)

Note

Abbreviations: ECOG, Eastern Cooperative Oncology Group; IQR, interquartile range; LUTS, lower urinary tract symptoms; PSA, prostate‐specific antigen; UICC, Union for International Cancer Control.

**FIGURE 2:**
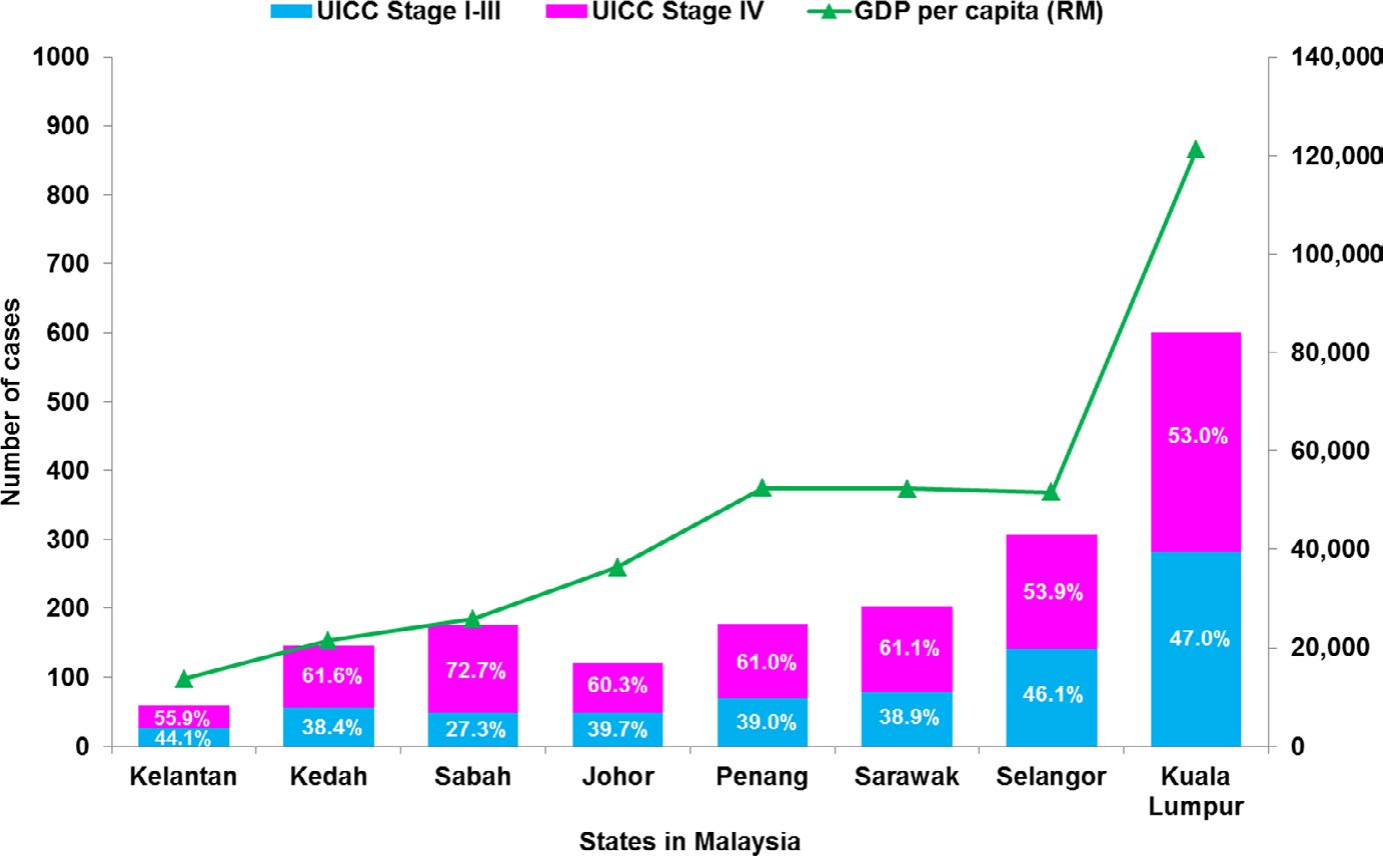
Distribution of prostate cancer new cases and gross domestic product (GDP) per capita across different states in Malaysia.

Approximately one third of the patients presented with PSA level of 20 ng/ml or less at diagnosis (median PSA: 54.85 ng/ml, IQR 15.77–228.29). Majority of the tumours (96.4%) were of adenocarcinoma histology; of which, 956 (54.8%) tumours exhibited Gleason score 8–10. Nearly three quarters of the patients were diagnosed with advanced stage disease including 14.2% with stage III and 58.1% with stage IV prostate cancer. Presence of metastasis (M1) was observed in 85.9% (892/1039) of stage IV tumours. Although prostate cancer was more common among Chinese ethnicity (45.2%), late‐stage prostate cancers (stage IV) were detected more frequently in Malays (62.5%), Indians (57%) and Sabah & Sarawak natives (77%) than in Chinese (50.2%) (*p* < 0.01, Chi‐square Test). In addition, we observed a negative trend between number of stage IV disease and gross domestic product (GDP) per capita of each state^12^ (Figure 2). For instance, a relatively higher percentage of stage IV disease were reported in urology referral centre located in Sabah (72.7%) with GDP per capita of ~US$ 6, 247 compared with those centres in Kuala Lumpur (stage IV cases: 53%; GDP per capita: ~US $ 29,298).^12^

Table 2 illustrates the primary treatment patterns based on disease stages. There were 297 patients undergoing radiotherapy; of which, 97% (288/297) received combined radiotherapy and ADT, whilst 3% (9/297) treated with radiotherapy alone. The latter had either localised disease (stage I = 2 & stage II = 4) or advanced disease (stage III = 2 and stage IV =1). For localised prostate cancer, active surveillance/watchful waiting was opted by 56.9% of stage I patients whilst the first‐line treatments for stage II patients were radiotherapy (either combined RT and ADT, 29.6% or RT alone, 1.5%) and radical prostatectomy (30.7%). Overall, most patients receiving surgical intervention underwent minimally invasive procedures including robot‐assisted radical prostatectomy (62.7%) and laparoscopic radical prostatectomy (8.5%), whilst the remaining 28.8% received retropubic radical prostatectomy. Notably, there are two surgical robot systems available at the public urology referral centres in Malaysia.

**TABLE 2:** Primary treatment patterns of newly diagnosed patients with prostate cancer based on disease stages.

UICC staging	Frequency distribution, n (%)				Total (N = 1615)
	Radical prostatectomy (n = 177)	Radiotherapy (n = 297)	Primary ADT (n = 987)	Active surveillance/watchful waiting (n = 154)	
Stage I	16 (11.7)	24 (17.5)	19 (13.9)	78 (56.9)	137 (8.5)
Stage II	79 (30.7)	80 (31.1)	53 (20.6)	45 (17.5)	257 (15.9)
Stage III	58 (25.6)	122 (53.7)	26 (11.4)	21 (9.3)	227 (14.1)
Stage IV	24 (2.4)	71 (7.1)	889 (89.4)	10 (1.0)	994 (61.5)

Note

Abbreviations: ADT, androgen deprivation therapy; UICC, Union for International Cancer Control.

Early stage prostate cancer patients (stage I and II) treated with ADT alone were significantly older with median age at diagnosis of 76 years (IQR 70–79.5) than those receiving combination of RT and ADT (median age at diagnosis: 70.5 years, IQR 67–73) and radical prostatectomy (median age at diagnosis: 67 years, IQR 64–70) (*p *< 0.01, Kruskal–Wallis test). Patients with locally advanced prostate cancer (stage III) were predominantly treated with combination of RT and ADT (52.9%). ADT was the primary treatment of stage IV patients (89.4%); of which, administration of chemohormonal therapy was observed in 4.7% (42/889) of patients. Gonadotrophin‐releasing‐hormone (GnRH) agonists or antagonists (76.5%) was the most common form of ADT and followed by orchidectomy (21.5%).

**FIGURE 3:**
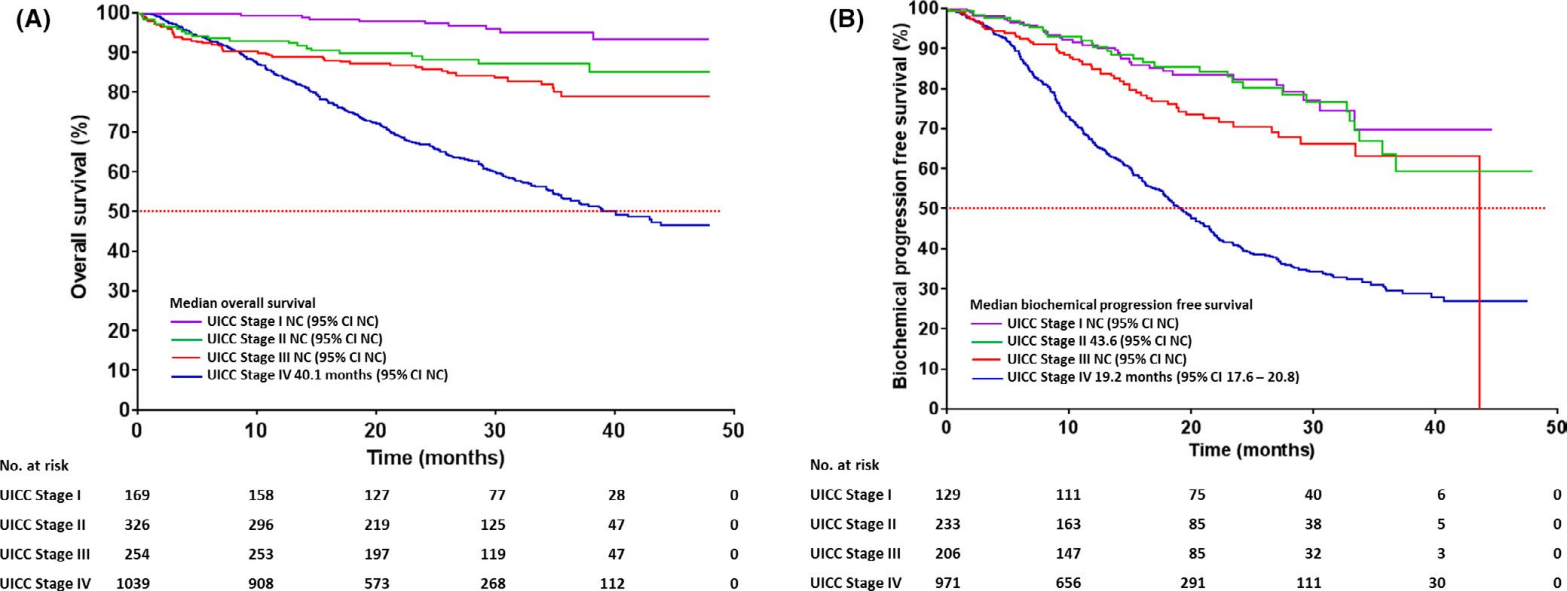
The (A) overall survival and (B) biochemical progression‐free survival of patients with M‐CaP stratified by disease stages.

A total of 490 deaths (26.6%) were recorded in the M‐CaP patients (N = 1839); of which, prostate cancer accounted for 48.6% (238/490) of mortality. The median follow‐up for all patients was 23.5 months (IQR 15.9–33.6 months). The median OS and bPFS of stage IV patients were 40.1 months and 19.2 months (95% CI 17.6–20.8), respectively (Figure 3). In addition, 22.5% (56/249) of metastatic patients received life‐prolonging treatments after developing biochemical progression or castration resistant prostate cancer (CRPC). These included chemotherapy (30.3%) and novel, androgen receptor‐targeting agents such as abiraterone acetate (64.3%) and enzalutamide (5.4%).

## 4 DISCUSSION

In this longitudinal observational study, we have shown that approximately three‐quarters of M‐CaP patients presented with advanced‐stage prostate cancer. This finding is consistent with previous reports showing that 68.6% of patients with prostate cancer were diagnosed with stage III and IV disease with an overall age‐standardised incidence rate of 7.7 per 100,000 population.^13^ We report for the first time the ethnic and geographic disparities in late‐stage (stage IV) prostate cancer diagnosis among multi‐ethnic Asian men at real‐world setting.

Prostate cancer is one of the most curable cancers in men if diagnosed and treated at an early stage. Evidence from the Prostate Testing for Cancer and Treatment (ProtecT) Trial showed that more than 90% of localised patients with prostate cancer survived at median of 10 years irrespective of their treatment modalities.^14^ Conversely, patients with late‐stage disease are with poorer prognosis and higher mortality‐to‐incidence ratio. Public awareness of prostate cancer and its disease progression plays a pivotal role in improving the outcome of patients with prostate cancer. Previous studies suggested that gaps in prostate cancer awareness and knowledge about PSA testing, disease prognosis as well as prostate cancer treatment may reflect different levels of education, socioeconomic status, healthcare provision and media coverage across various populations.^15^, ^16^ It has also been shown that the prostate cancer mortality‐to‐incidence ratio was significantly lower in countries with high GDP and healthcare expenditure than those of middle‐to‐low income countries.^17^, ^18^ These findings may explain the ethnic and geographic disparities of late stage cancer in the M‐CaP cohort. For instance, hospitals in states with higher GDP per capita such as Kuala Lumpur and Selangor, albeit with higher number of new cases, had a lower number of stage IV cases compared with those of states with lower GDP per capita (Figure 2). Significant discrepancies of late‐stage disease were also observed across different ethnicities in the M‐CaP patients, suggesting low level of awareness and poor understanding of prostate cancer^19^ together with limited access to healthcare may delay early diagnosis of prostate cancer.

In addition, there are some variations of localised prostate cancer treatment patterns among M‐CaP patients compared with other Asian countries. For instance, almost a third of M‐CaP patients diagnosed with localised disease underwent active surveillance, whilst 24% underwent radical prostatectomy. Conversely, most localised Japanese patients preferred to receive some forms of treatments including robotic‐assisted radical prostatectomy, hormonal therapy or radiotherapy, which are approved under the Japanese healthcare insurance system.^8^ Active surveillance was opted by minority of the Japanese patients only.^8^ To date, more than 900 da Vinci surgical robot systems have been installed across Japan, representing one of largest numbers worldwide.^20^ Similar treatment trend was observed in Korean men.^8^

The documented evidence of prostate cancer survival analyses is scarce in this region. The Malaysian Study on Cancer Survival (MySCan) reported the median survival time of prostate cancer was 58.02 months (95% CI 56.62–61.73), whilst the 5‐year relative survival of stages I,II, III and IV prostate cancer was 97.3%, 92.1%, 93.0% and 43.2%, respectively.^21^ These findings were based on prostate cancer diagnosed between 2007 and 2011 with a minimum follow‐up of 5 years until 2016. Comparing with the M‐CaP cohort, the median OS of stage IV prostate cancer (40.1 months) only was defined at this stage because the number of deaths from other disease stages was less than 50%. It is worth noting that more robust analyses can be performed for 5‐year median OS and PFS when the median follow‐up of M‐CaP database reaches 5 years and above. In addition, our survival data revealed that there were 12.9% of prostate cancer‐specific deaths at 23.5 months median follow‐up, attributing to advanced disease and limited access to survival‐prolonging treatments. In the M‐CaP cohort, only 22.5% of patients with metastatic CRPC received life‐prolonging treatments as most patients cannot afford abiraterone (~US$ 2800/month) or enzalutamide (~US$ 3400 for 28 days), that is more than twofold higher than their median monthly household income (~US$ 1410).^22^, ^23^ Personal insurance coverage is affordable by 18% of Malaysian patients, particularly those in the high‐income groups only.^24^ Recent findings from the ACTION (ASEAN Costs in Oncology) study showed that 48% of cancer patients experienced financial catastrophe (out‐of‐pocket health costs ≥30% of annual household income) whilst 45% underwent economic hardship (inability to make necessary household payments) following a 12‐month cancer diagnosis in Malaysia.^24^ These adverse financial catastrophes were mainly resulted from medical expenses for inpatient/outpatient care, drugs, medical supplies and equipment. To overcome this economic hardship, 28% of affected families took personal loans, and 60% used savings that were previously set aside for other uses.^24^

There are limitations of this M‐CaP database. First, the data are observational and non‐randomised in nature. Selection biases and unmeasured confounding variables may affect the validity of the results,^25^ although these issues can be partly addressed by several statistical modelling techniques. Second, addition of private urology referral centres may increase the number of patients in the database. However, that will require substantial financial and human resources, which are ongoing challenges in sustaining most disease‐specific registries. Third, tissue‐ or serum‐based marker database is currently unavailable in the M‐CaP cohort. This pitfall should be carefully considered in the future studies as biomarker investigations might improve our understanding of the natural history of prostate cancer particularly in our population.

The strength of this M‐CaP cohort are the large sample size (>1000 subjects) and adequate representation from three major Asian ethnicities. To our knowledge, this is the first longitudinal multicentre study assessing the presentation, management and survival following prostate cancer in multi‐ethnic Asian men. There is a low rate of missing data in most clinical variables ranged 1.3–12.2% except family history of prostate cancer, owing to more sensitive nature of personal family medical history. This prospective database can be utilised to investigate potential risk factors and prognostic factors associated with incidence and mortality rates, based on patient characteristics such as age, race, socioeconomic status, comorbidity burden and disease stage. Trends of treatment modalities and novel drug adoption can be evaluated in relation to disease outcome from this database at real‐world setting.

## 5 CONCLUSION

In summary, we conclude that late stage at presentation remains a challenge in multi‐ethnic Asian men, posing tremendous pressure to the existing healthcare system. Evidence‐based, holistic cancer control strategies are crucial to encourage early detection and reduce the burden of advanced prostate cancer in this part of the world.

## CONFLICT OF INTEREST

All authors declare no conflict of interest.

## ETHICAL APPROVAL

Ethical approval for the M‐CaP database was granted by the Medical Research and Ethics Committee (MREC), Ministry of Health Malaysia and UMMC. Informed consent was exempted by the medical research ethics committee due to the adoption of secondary data collection methods.

## Supporting information

Data S1

## Data Availability

The data used to support the findings of this study are included in the article.

## References

[1] GLOBOCAN 2020: Estimated cancer incidence, mortality and prevalence worldwide in 2020. https://gco.iarc.fr/today/home. Accessed October 5, 2021.

[2] Lim J , Onozawa M , Saad M , et al. Recent trend of androgen deprivation therapy in newly diagnosed prostate cancer patients: Comparing between high‐ and middle‐income Asian countries. Cancer Sci. 2021;112:2071‐2080. 33738901 10.1111/cas.14889 PMC8177804

[3] Farmer P , Frenk J , Knaul FM , et al. Expansion of cancer care and control in countries of low and middle income: a call to action. Lancet. 2010;376:1186‐1193. 20709386 10.1016/S0140-6736(10)61152-X

[4] Matsuoka T , Kawai K , Kimura T , et al. Long‐term outcomes of combined androgen blockade therapy in stage IV prostate cancer. J Cancer Res Clin Oncol. 2015;141:759‐765. 25326347 10.1007/s00432-014-1856-3 PMC11823761

[5] Liu Y , Uemura H , Ye D , et al. Prostate cancer in Asia: design of a patient registry to inform real‐world treatments, outcomes, and quality of life. Prostate Int. 2019;7:108‐113. 31485435 10.1016/j.prnil.2018.12.001 PMC6713796

[6] Akaza H , Hirao Y , Kim C‐S , et al. Asia prostate cancer study (A‐CaP Study) launch symposium. Prostate Int. 2016;4:88‐96. 27689065 10.1016/j.prnil.2016.03.001 PMC5031897

[7] Kim C‐S , Lee JY , Chung BH , et al. Report of the Second Asian Prostate Cancer (A‐CaP) study meeting. Prostate Int. 2017;5:95‐103. 28828352 10.1016/j.prnil.2017.03.006 PMC5551923

[8] Lojanapiwat B , Lee JY , Gang Z , et al. Report of the third Asian Prostate Cancer study meeting. Prostate Int. 2019;7:60‐67. 31384607 10.1016/j.prnil.2018.06.001 PMC6664304

[9] Youl Lee JI , Taniguchi T , Zhang K , et al. Report of the forth Asian Prostate Cancer (A‐CaP) study meeting. Jpn J Clin Oncol. 2019;49:581‐586. 31141613 10.1093/jjco/hyz053

[10] EAU‐EANM‐ESTRO‐ESUR‐SIOG Guidelines on Prostate Cancer . 2020. https://uroweb.org/wp‐content/uploads/EAU‐EANM‐ESTRO‐ESUR‐ISUP‐SIOG‐Guidelines‐on‐Prostate‐Cancer‐2021V3.pdf. Accessed October 5, 2021.

[11] Roach M , Hanks G , Thames H , et al. Defining biochemical failure following radiotherapy with or without hormonal therapy in men with clinically localized prostate cancer: recommendations of the RTOG‐ASTRO Phoenix Consensus Conference. Int J Radiat Oncol Biol Phys. 2006;65:965‐974. 16798415 10.1016/j.ijrobp.2006.04.029

[12] Department of Statistics Malaysia Official Portal: State Socioeconomic Report. 2018. https://www.dosm.gov.my/v1/index.php?r=column/cthemeByCat&amp;cat=102&amp;bul_id=a0c3UGM3MzRHK1N1WGU5T3pQNTB3Zz09&amp;menu_id=TE5CRUZCblh4ZTZMODZIbmk2aWRRQT09. Accessed October 5, 2021.

[13] Malaysia National Cancer Registry Report . 2012–2016. https://www.moh.gov.my/moh/resources/Penerbitan/Laporan/Umum/2012‐2016%20(MNCRR)/MNCR_2012‐2016_FINAL_(PUBLISHED_2019).pdf. Accessed October 5, 2021.

[14] Hamdy FC , Donovan JL , Lane JA , et al. 10‐Year outcomes after monitoring, surgery, or radiotherapy for localized prostate cancer. N Engl J Med. 2016;375:1415‐1424. 27626136 10.1056/NEJMoa1606220

[15] Schulman CC , Kirby R , Fitzpatrick JM . Awareness of prostate cancer among the general public: findings of an independent international survey. Eur Urol. 2003;44:294‐302. 12932926 10.1016/s0302-2838(03)00200-8

[16] Akakura K , Bolton D , Grillo V , Mermod N . Not all prostate cancer is the same – patient perceptions: an Asia‐Pacific region study. BJU Int. 2020;126(Suppl 1):38‐45. 32521568 10.1111/bju.15129

[17] Batouli A , Jahanshahi P , Gross CP , Makarov DV , Yu JB . The global cancer divide: relationships between national healthcare resources and cancer outcomes in high‐income vs. middle‐ and low‐income countries. J Epidemiol Glob Health. 2014;4:115‐124. 24857179 10.1016/j.jegh.2013.10.004 PMC7366371

[18] Neupane S , Bray F , Auvinen A . National economic and development indicators and international variation in prostate cancer incidence and mortality: an ecological analysis. World J Urol. 2017;35:851‐858. 27744614 10.1007/s00345-016-1953-9

[19] Muhammad Fadhil Hafiz MS , Soon LK , Azlina Y . Knowledge, awareness and perception towards prostate cancer among male public staffs in Kelantan. Int J Public Health Clin Sci. 2016;3:105‐115.

[20] Japan Robotic Surgery Society . Key statistics of da Vinci system in Japan. 2020. https://j‐robo.or.jp/robot/da‐vinci/jisseki.html. Accessed October 5, 2021.

[21] Malaysian Study on Cancer Survial (MySCan) . https://www.moh.gov.my/moh/resources/Penerbitan/Laporan/Umum/Malaysian_Study_on_Cancer_Survival_MySCan_2018.pdf. Accessed October 5, 2021.

[22] Department of Statistics Malaysia Official Portal: Key Statistics of Household Income and Expenditure 2019 . https://www.dosm.gov.my/v1/index.php?r=column/cthemeByCat&amp;cat=120&amp;bul_id=TU00TmRhQ1N5TUxHVWN0T2VjbXJYZz09&amp;menu_id=amVoWU54UTl0a21NWmdhMjFMMWcyZz09. Accessed October 5, 2021.

[23] Saad M , Alip A , Lim J , et al. Management of advanced prostate cancer in a middle‐income country: real‐world consideration of the Advanced Prostate Cancer Consensus Conference 2017. BJU Int. 2019;124:373‐382. 31077523 10.1111/bju.14807 PMC6851975

[24] ACTION Study Group . Policy and priorities for national cancer control planning in low‐ and middle‐income countries: Lessons from the Association of Southeast Asian Nations (ASEAN) Costs in Oncology prospective cohort study. Eur J Cancer. 2017;74:26‐37. 10.1016/j.ejca.2016.12.014 28335885

[25] Giordano SH , Kuo YF , Duan Z , Hortobagyi GN , Freeman J , Goodwin JS . Limits of observational data in determining outcomes from cancer therapy. Cancer. 2008;112:2456‐2466. 18428196 10.1002/cncr.23452 PMC3856661

