# Supplementary material for: Prostate cancer in multi‐ethnic Asian men: Real‐world experience in the Malaysia Prostate Cancer (M‐CaP) Study
Source: Cancer Med. 2021 Oct 9;10(22):8020–8. doi: 10.1002/cam4.4319 (PMC8607241; doi:10.1002/cam4.4319)
Supplement: Supplementary file 1 — Data S1 [file CAM4-10-8020-s001.docx]

**Supplementary Note**

M-CaP Study (in addition to those named in the author list)

Shakirin Kamaruzaman^1^, Chai Woon Gan^1^, Sarah Qian Rou Choo^1^, Hong Xian Hoe^1^, Zurina Che Rohani^1^, Seow Huey Choy^1^

^1^Department of Surgery, Faculty of Medicine, University of Malaya, Kuala Lumpur, Malaysia.
